# First Report on the Emergence of *Neopestalotiopsis rosae* as a Severe Economic Threat to Strawberry Production in Germany

**DOI:** 10.3390/microorganisms13010006

**Published:** 2024-12-24

**Authors:** Tom E. Schierling, Ralf T. Voegele, Abbas El-Hasan

**Affiliations:** Department of Phytopathology, Institute of Phytomedicine, Faculty of Agricultural Sciences, University of Hohenheim, Otto-Sander-Str. 5, D-70599 Stuttgart, Germany; ralf.voegele@uni-hohenheim.de

**Keywords:** strawberry, first report, fungal pathogen, Koch’s postulates, PCR, sequencing, pathogenicity, phylogeny, morphological assessment

## Abstract

Strawberries hold significant economic importance in both German and global agriculture. However, their yield is often adversely affected by fungal diseases. This study describes *Neopestalotiopsis rosae* as a newly emerging pathogen responsible for leaf blight and fruit rot in strawberries in Germany. Infected plants were observed in Hohenheim, Germany. A combination of morphological and molecular analyses, along with pathogenicity tests, confirmed the identity of *N. rosae* as the causal agent. Morphological examination of conidia and mycelium revealed key characteristics including the presence of versicolorous median cells, conidial appendages, black spherical conidiomata formation as well as changing colony color and fluffy texture. These properties align with the established descriptions for the species. Molecular analysis, particularly the sequencing of the internal transcribed spacer and β-tubulin regions allowed the precise identification of the pathogen. Artificial inoculation of healthy strawberry plants with conidial suspension derived from the isolated strain resulted in the development of characteristic symptoms, including necrotic leaf spots and water-soaked fruit lesions, similar to those observed on the original infected plants. To our knowledge, this study presents the first documented occurrence of *N. rosae* in Germany, highlighting its emergence as a significant threat to strawberry production in Europe.

## 1. Introduction

Strawberry (*Fragaria* × *ananassa* Duch.) is frequently affected by various pathogens that reduce both yield and quality, resulting in significant economic losses [1]. These pathogens can cause a range of severe diseases, including leaf spot (*Mycospaerella fragariae*), fruit and rhizome rot (*Botrytis* sp., *Phytophthora* spp. and *Collototrichum* sp.), and wilt (*Fusarium* spp. and *Verticillium* sp.), which not only reduce yield quantitively, but also have a negative impact on fruit quality, rendering fruits unsuitable for the market [1,2,3,4]. Fungal pathogens force growers to increase agricultural inputs, i.e., fungicides and labor, thereby raising production costs and complicating agricultural practices [5]. Additionally, emerging fungal pathogens like *Pestalotia* spp. have been increasingly recognized as severe threats in several strawberry producing countries including India, Israel, Egypt, the United States, and Brazil [6,7,8,9,10]. This highlights the widespread challenges growers face in managing these diseases effectively.

*Pestalotia* is an outdated genus name, and fungi previously grouped under this genus have been re-classified under various genera such as *Pestalotiopsis*, *Pseudopestalotiopsis*, and *Neopestalotiopsis*, among others [11]. In general, members belonging to the genus *Pestalotiopsis*, including *Neopestalotiopsis* spp., have been known to cause diseases in a wide range of host plants, including strawberry [12,13,14]. These diseases often lead to symptoms such as leaf spots, fruit rot, and blight, and can be significant in humid, warm climates that favor fungal growth [15]. Reports from various regions indicate that related pathogens were associated with strawberry production across various countries.

In Mexico, *Neopestalotiopsis rosae* has been identified as a causal agent of root rot, crown rot, and leaf spot in strawberries, marking the first report of this species infecting strawberries worldwide [15]. At the same time, *N. rosae* has been reported to cause leaf blight and crown rot in Taiwan [16]. In South America, *Neopestalotiopsis clavispora* has been implicated in root and crown rot diseases in countries such as Uruguay and Argentina indicating the presence of multiple *Neopestalotiopsis* species affecting strawberry crops in this region [17,18]. Furthermore, *Neopestalotiopsis mesopotamica* has been reported in Ecuador, marking its first association with strawberry crown rot [19]. In the United States, particularly in Florida, *Neopestalotiopsis* spp. have been identified as significant contributors to leaf spot and fruit rot diseases in strawberries [20,21].

The emergence of a new *Neopestalotiopsis* species in Florida has raised alarms among growers, as this species appears to be more aggressive and destructive compared to previously known pathogens. Genetic analyses of isolates from Florida have shown low genetic diversity among them, yet a clear separation into distinct groups, indicating localized adaptations [20]. This highlights the importance of understanding the genetic diversity and pathogenicity of *Neopestalotiopsis* species to inform management practices. In Asia, while *Neopestalotiopsis chrysea* causing leaf spot disease in strawberries was reported in Bangladesh, various *Pestalotiopsis* species have been associated with crown rot diseases in Vietnam [22]. In China, *N. rosae* has been identified to cause root rot in strawberries [23]. Recently, the pathogen has been reported to cause leaf blight and crown rot on strawberries in India [24]. In Europe, *N. clavispora* was reported to cause root and crown rot in strawberries in Spain and Italy [25,26]. However, *N. rosae* has not yet been reported in Europe. The rapid occurrence and spread of various *Neopestalotiopsis* species across countries and continents in the past decade is an alarming threat to global strawberry production, especially since there are little to no chemical options for their management [21].

*N. rosae* and *N. clavispora* can be considered the most reported *Neopestalotiopsis* species on strawberries in the world. The symptoms incited by *N. rosae* are mainly leaf spot and crown rot, but in some cases root rot was also observed [20,23,27]. In contrast, *N. clavispora* primarily causes root rot in strawberries, making correct identification and differentiation from another species essential for effective management [17,18,25,26]. Furthermore, symptoms on leaves and fruit caused by *Neopestalotiopsis* spp. can be easily confused with those of other fungal pathogens like *Colletotrichum* sp., *Fusarium* sp., *Verticillium* sp., *Phytophthora* sp., and *Pestalotiopsis* sp., all of which can lead to wilting, leaf blight, and fruit and root rot on strawberries [28,29,30,31,32].

Given the serious threats to strawberry cultivation, the emergence of a new fungal pathogen like *N. rosae* increases the need for reliable pathogen identification and differentiation. This study focuses on the emerging fungal disease, recently observed to cause leaf blight and fruit rot on strawberry plants in Germany. The primary objectives were to isolate the pathogen from infected plant material, carry out comprehensive morphological and molecular analyses, and perform pathogenicity tests to confirm Koch’s postulates, thereby confirming the causal relationship between the isolated pathogen and the observed disease symptoms.

## 2. Materials and Methods

### 2.1. Sample Collection and Pathogen Isolation

During the 2023 growing season, strawberry leaves of the cultivar Aprica with wilting symptoms and necrosis were collected from a field trial at the Agricultural Research Station location Heidfeldhof (University of Hohenheim, Stuttgart, Germany). Upon collection, samples were transported to the laboratory in breathable bags to minimize moisture accumulation and were processed within 24 h. To eliminate potential surface contaminants, detached leaves were subjected to surface sterilization using 1% sodium hypochloride for 1 min and subsequently rinsing in sterile water. Leaves were then incubated for 5 days in a humidity chamber. The humidity chambers were sealed airtight with Parafilm and incubated at 21 ± 2 °C and 16/8 h (light/dark) cycle. Regular observations were made to monitor the development of mycelium and conidia.

After incubation, fungal colonies that developed from the tissue were subcultured onto Glucose-Medium-7-Agar (GM7) [33] to obtain pure fungal cultures. After 14 days of incubation, conidia were obtained from the cultures with a sterile pipette and dispersed in 500 µL of sterile water. The conidial suspension was mixed vigorously, spread onto a new Petri dish containing a thin film of GM7. Inoculated cultures were sealed airtight with Parafilm and incubated at a 16/8 h light/dark cycle for 24 h. Subsequently, a sterile needle was used to cut out small pieces of GM7, containing one germinated conidia each. Cut agar pieces with one conidia were transferred to new GM7 petri dishes and incubated for 14 days. The resulting single spore culture was designated as AETS11.

### 2.2. Morphological Characterization

**Figure 1:**
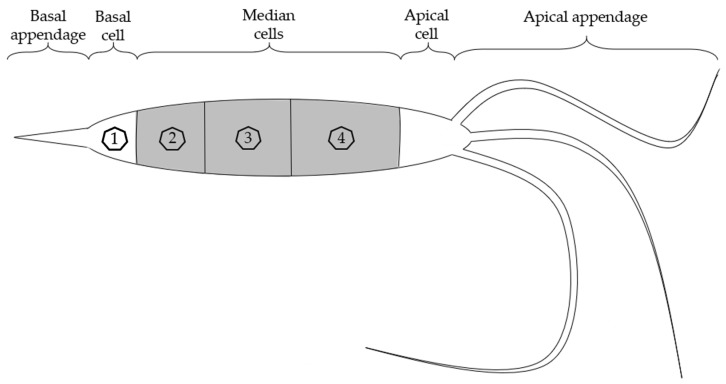
Schematic illustration of *Neopestalotiopsis rosae* conidia with individual cells. Numbers represent first to fourth cell from the base.

Colony formation and conidial characteristics were recorded after 14 days. Colony and conidiomata color, shape, and formation were determined using a binocular (Zeiss, Oberkochen, Germany). The mean length, number of septa, length of appendages, as well as the basal, second to fourth cell from the base, median, and apical cells of 30 conidia were determined using the AxioVision release 4.6.3 software and an AxioCam MRm monochromatic camera, attached to an optical microscope Axioskop 2 (Zeiss) (Figure 1).

### 2.3. Pathogenicity Test

To evaluate the pathogenicity of the isolated fungal strain AETS11 on strawberry plants, a greenhouse experiment was conducted. A conidial suspension was made by adding 4 mL of 0.01% Tween solution to a 14-day-old culture of AETS11. Subsequently, the fungal material was gently scraped off with a spatula. The conidial suspension was filtered through four layers of sterile gauze (Hartmann, Heidenheim, Germany). The concentration of conidia was measured using a Fuchs–Rosenthal counting chamber (Brand, Wertheim, Germany) and adjusted to 10^5^ conidia mL^−1^. Five healthy strawberry seedlings (cv. Sengana) with three replicates (15 plants in total) were sprayed with the conidial suspension of AETS11 using a chromatography sprayer until runoff (~2 mL each). A total of 15 control plants were sprayed with the same amount of 0.01% Tween solution. To maintain an appropriate relative humidity, plants were placed on fleece saturated with water and covered with translucent plastic boxes. At 24 h intervals, boxes were removed for 8 h. Plants were incubated for 21 days at 21 ± 2 °C under 16/8 h light/dark conditions. Plants were examined for disease symptoms. To monitor the development of fruit rot symptoms on incubated fruit, fruits developed during this experiment were detached when fully ripened and stored in a humidity chamber, made of sterile plastic boxes, filled with one layer of wetted sterile paper tissue.

### 2.4. Re-Isolation of Fungal Strains from Greenhouse Plants

To fulfill Koch’s postulates, symptomatic leaves from the pathogenicity test were detached after 21 days and the pathogen was isolated as described in Section 2.1. This re-isolated fungal strain was named AETS11R. Additionally, rhizomes from infected plants were thoroughly rinsed with sterile water to remove the soil from their surface. Subsequently, plant rhizomes were cut lengthwise and examined for the presence of discoloration lesions or other symptoms associated with infection. The inner tissue was then cultured on GM7 agar to confirm the presence of the fungal pathogen and to assess its characteristics. This step ensured that the re-isolated strain matched the original isolate, thereby validating the results of the pathogenicity test.

### 2.5. DNA Extraction, PCR Amplification, and Sequencing

Fungal mycelium was scraped off from 14-day-old cultures of AETS11 and AETS11R and extracted using the rapid mini preparation of fungal DNA for PCR method [34]. For the amplification of the Internal Transcribed Spacers (ITS) and β-Tubulin (TUB) regions, primers ITS5/ITS4 (GGAAGTAAAAGTCGTAACAAGG/TCCTCCGCTTATTGATATGC) [35] and BT2a/BT2b (GGTAACCAAATCGGTGCTGCTTTC/ACCCTCAGTGTAGTGACCCTTGGC) [36,37] were used, respectively.

For PCR, the following components were used: 8 µL 5× HF Phusion buffer (ThermoFisher, Sindelfingen, Germany), 0.8 µL deoxynucleoside triphosphate mix (dNTPs) at 10 µM, 1 µL of each primer at 10 pM, 0.4 µL Phusion Polymerase at 2 U µL^−1^ (ThermoFisher, Waltham, MA, USA), 1 µL of DNA template at 7.5 ng µL^−1^, and 27.8 µL of purified water. PCR conditions for the ITS region were as follows: an initial denaturation at 98 °C for 30 s, followed by 35 cycles of denaturation at 98 °C for 10 s, annealing at 55 °C for 15 s, extension at 72 °C for 35 s, and a final extension at 72 °C for 10 min. PCR conditions used for the amplification of TUB gene were the following: an initial denaturation at 98 °C for 30 s, followed by 35 cycles of denaturation at 98 °C for 5 s, annealing at 55 °C for 15 s, extension at 72 °C for 45 s, and a final extension at 72 °C for 5 min. PCR products were visualized on 2% agarose gels, purified using the peqGOLD Cycle-pure-kit (Peqlab, Erlangen, Germany), and sent for sequencing (Mycrosynth Seqlab, Göttingen, Germany). Sequences were deposited to the National Library of Medicine (NCBI) GenBank database (Appendix A, Table A1) [38].

### 2.6. Sequence Alignment and Phylogenetic Analysis

For phylogenetic analyses, sequences of ITS and TUB regions were chosen according to Maharachchikumbura et al. (2014), Norphanphoun et al. (2019), and Sun et al. (2021) (Appendix A, Table A1) [11,23,39]. Sequences were downloaded from the NCBI GenBank database. ITS and TUB sequences were condensed with BioEdit (version 5.0.9) and aligned using the ClustalW algorithm in the MEGA software (version 11) [40]. Alignments were edited with BioEdit (version 5.0.9) to remove incomplete and corrupted regions. Maximum likelihood (ML), maximum parsimony (MP), and Bayesian inference (BI) analyses were performed. *Pestalotiopsis diversiseta* (MFLUCC 12–0287) was used as the outgroup for the phylogenetic trees [23].

### 2.7. Evolutionary Analysis by Maximum Likelihood Method

Evolutionary history was inferred by using the Maximum Likelihood and the Hase-Gawa–Kishino–Yano (HKY) model [41]. The HKY model was determined as the best-fit model with the Jmodeltest (version 2) in the PAUP software (version 4). Initial tree(s) for the heuristic search were obtained automatically by applying Neighbor-Join and BioNJ algorithms to a matrix of pairwise distances estimated using the Maximum Composite Likelihood (MCL) approach, and then selecting the topology with superior log likelihood value. A discrete Gamma distribution was used to model evolutionary rate differences among sites (five categories (+*G*, parameter = 0.4667)). The rate variation model allowed for some sites to be evolutionarily invariable (+*I*, 36.51% sites). A total of 1000 bootstrap replicates were developed. Evolutionary analyses were conducted using MEGA software (version 11).

### 2.8. Maximum Parsimony Analysis of Taxa

Evolutionary history was also inferred using the Maximum Parsimony method. The MP tree was obtained using the Tree-Bisection-Regrafting (TBR) algorithm with search level 1 in which the initial trees were obtained by the random addition of sequences (10 replicates) [42]. A total of 1000 bootstrap replicates were developed. Evolutionary analyses were conducted in MEGA software (version 11).

### 2.9. Bayesian Inference Analysis

A Bayesian Inference analysis was performed based on the Markov Chain Monte Carlo (MCMC) method using the MrBayes software (version 3.81) [43]. The HKY + G + I model was selected as the best-fit model for the combined gene expression analysis with the MrModeltest software (version 2). For combined sequences, the MCMC method was run for 2,000,000 generations with four chains, starting from a random tree topology. Trees were sampled every 100 generations. Phylogenetic trees were opened with FigTree (version 1.4.4) and edited in MS PowerPoint.

## 3. Results

### 3.1. Symptoms of N. rosae on Naturally Infected Strawberry Plants

**Figure 2:**
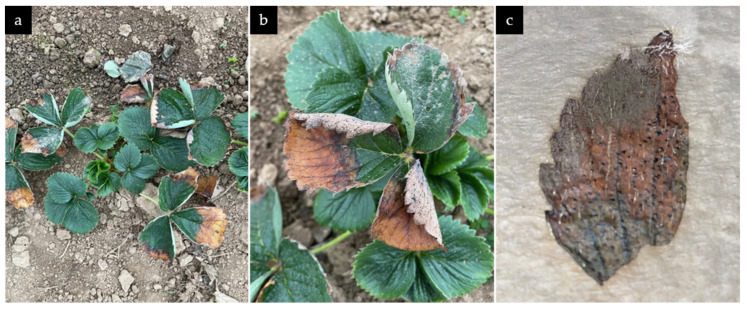
(**a**,**b**) Necrosis, leaf curling, burning, and conidiomata on naturally infested plants in the field trial; (**c**) conidiomata and mycelia on detached leaf in humidity chamber.

Several leaves with necrotic spots on the edges were observed shortly after planting strawberry cv. Aprica (Figure 2a). Starting from the edges, and in later stages commencing to the leaf center, yellow to orange and eventually brown to black necrotic spots were formed. Additionally, leaves started rolling from the edges. Further development showed the formation of black conidiomata on the leaf surface (upper and under side) with a greasy consistency (Figure 2b). Detached leaves in humidity chambers showed further necrosis, formation of white mycelia, and black conidiomata (Figure 2c). Lesions on the fruit started with soft tissue, subsequently forming sunken wet spots and ultimately covered in white mycelia with black structures (conidiomata).

### 3.2. Morphological Characterization

**Figure 3:**
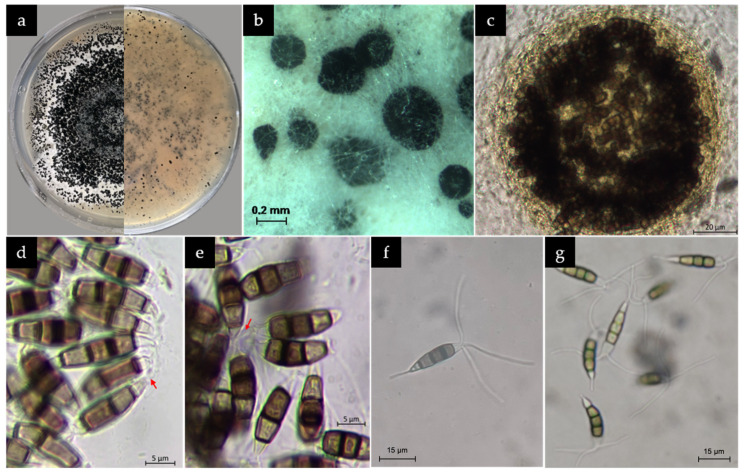
Various fungal structures used for the microscopic identification of *Neopestalotiopsis rosae*. (**a**) Growth on GM7 medium after 14 days. Left: Plate upper side. Right: Plate underside. (**b**,**c**) Conidiomata on agar. (**d**,**e**) Conidiophores (conidia producing structures, red arrows) (**f**,**g**) Conidia with characteristic versicolorous median cells and appendages.

Isolated colonies on GM7 demonstrated robust mycelial growth, with conidia formation observed after 14 days of incubation. Acervuli were not formed. Fungal colonies exhibited distinctive morphology, displaying circular growth, that initially appeared white and gradually darkened in color. The plate underside displayed a pale orange coloration. The texture of the colonies ranged from velvety to fluffy (Figure 3a).

Microscopic examination of the fungal colonies revealed the presence of typical features consistent with those observed in *Neopestalotiopsis rosae* [23]. While the sexual morph was not observed on agar, the asexual morph displayed conidiomata that were solitary, spherical to asymmetric, black, and contained glistening conidial masses (Figure 3b,c). Conidiophores were observed as conidiogenous cells (Figure 3d,e).

Conidia were observed to be fusoid or ellipsoidal to cylindrical in shape, straight or slightly curved, typically consisting of five cells. The central three cells were melanized, making them visibly darker (Figure 3f,g). The three middle cells were 15.78 ± 1.01 µm long ($\bar{x}$ ± SD, n = 30). The second cell from the base was hyaline to light grey or brown in color, 5.41 ± 0.63 µm long; the third cell was dark brown to black, 4.77 ± 0.62 µm long; and the fourth cell was grey to dark brown in color, 5.60 ± 0.8 µm long. The Basal cell was 4.60 ± 0.65 µm long, hyaline, conical, and often exhibited one basal appendage, hyaline, unbranched, emerging central from the very tip, and 7.86 ± 1.31 µm long. The Apical cell was 4.39 ± 0.63 µm long, conical, hyaline and exhibited two to four filiform, hyaline, unbranched appendages, 30.33 ± 4.2 µm long.

### 3.3. Pathogenicty Test and Re-isolation of N. rosae from Inoculated Plant Material

**Figure 4:**
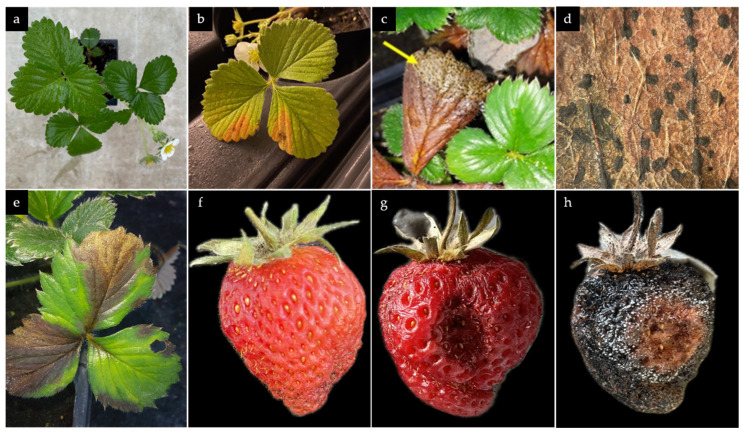
(**a**) Healthy, uninoculated control plant; (**b**) wilting and leaf spot formation on inoculated plant 14 dpi; (**c**,**d**) Conidiomata formation on inoculated, withered leaf 21 dpi; (**e**) V-shaped necrotic area on inoculated infected leaf; (**f**–**h**) fruit rot development on detached fruit test in a humidity chamber (1, 14, 21 dpi, respectively).

At 21 days post inoculation (dpi), uninoculated plants remained healthy (Figure 4a). Plants inoculated with *N. rosae* showed leaf spots and necrotic lesions, typically V-shaped and starting from the edges, which were later accompanied by conidiomata formation (Figure 4b–e). These symptoms closely resembled those initially observed on the source plants. Additionally, dark lesions on the fruit surface were observed, which typically began as small, water-soaked areas that gradually expanded and darkened, resulting in sunken, necrotic spots (Figure 4f,g). Furthermore, white mycelium covered the whole fruit and ultimately black conidiomata containing spore masses were formed (Figure 4h). A lengthwise cut through the plant rhizome did not reveal any symptoms on inner tissue. Roots were partly darkened. Cultivated root and rhizome tissue on GM7 did not show any fungal growth. Re-isolation and identification of the fungus from conidiomata formed on leaves and fruits confirmed it identity as *N. rosae*, thus establishing the causal link between the isolated fungus and the observed disease symptoms.

### 3.4. Phylogenetic Analysis

**Figure 5:**
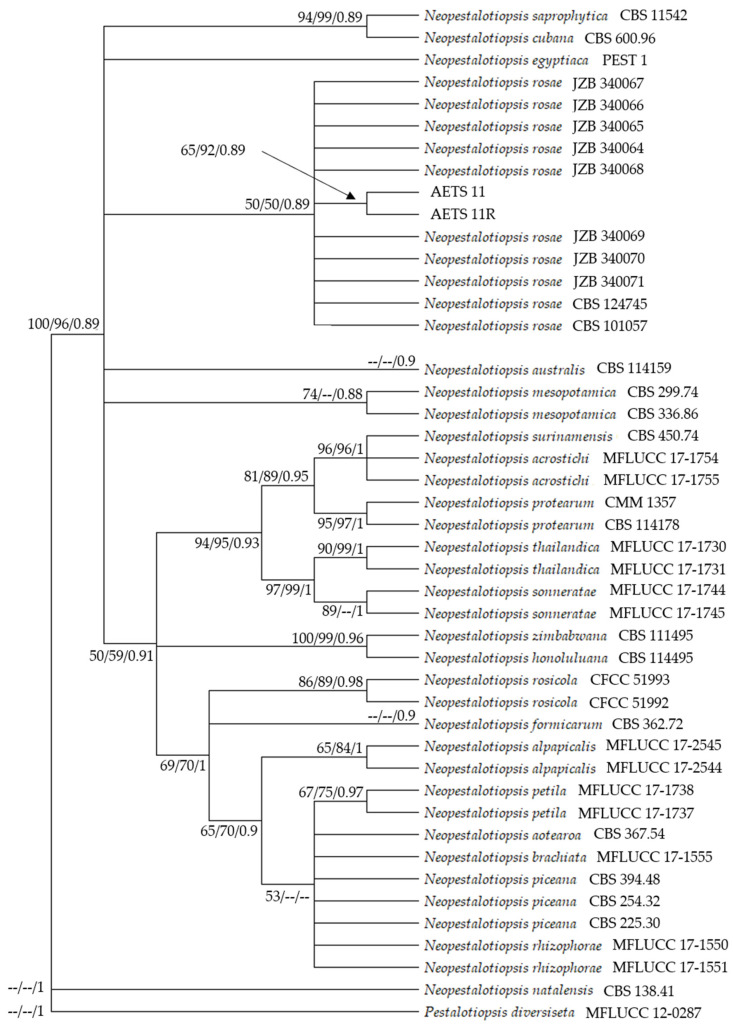
Phylogenetic tree resulting from ML analysis of the combined ITS and TUB dataset. ML and MP bootstrap support values (BS) greater than 50% and the Bayesian posterior probabilities (PP) greater than 0.85 are shown at the nodes. *Pestalotiopsis diversiseta* (MFLUCC 12–0287) was used as an outgroup.

The phylogenetic tree comprised 45 nucleotide sequences (taxa), with the outgroup *P. diversiseta* (MFLUCC 12–0287). The combined sequence alignment comprised both the ITS and TUB region. There was a total of 739 positions in the final dataset. The ML tree with the highest log likelihood (−1942.05) is shown (Figure 5).

The percentage of trees in which the associated taxa clustered together is shown next to the branches. ML and MP bootstrap values equal to or greater than 50% are shown in the ML tree. MP Tree one out of three most parsimonious trees (length = 233) was used. The consistency index was 0.884120 (0.640000), the retention index was 0.892000 (0.892000), and the composite index was 0.788635 (0.570880) for all sites and parsimony-informative sites (in parentheses).

The percentage of replicate trees in which the associated taxa clustered together in the bootstrap test (1000 replicates) are shown next to the branches. BI analysis resulted in a tree with the same topology and clades as the ML and MP trees. Phylogenetic analysis proved the strains AETS11 and AETS11R to be *N. rosae*. The ITS and TUB sequences from AETS11 and AETS11R have been submitted to the NCBI database (Appendix A, Table A1).

## 4. Discussion

The identity of *N. rosae* was confirmed through a combination of morphological identification and molecular phylogenetic analysis. Its pathogenicity was demonstrated on strawberry plants. The morphological characteristics of *N. rosae*, such as the versicolorous median cells and multiple appendages of its conidia, are consistent with those described for other strains within *N. rosae* [16,23]. When relying solely on ITS sequences, clades in the phylogenetic analysis remained uncertain and yielded inconsistent results across different phylogenetic analyses, which is in line with previous studies [11,23,44]. However, molecular analyses incorporating both ITS and TUB sequences provided additional confirmation of the pathogen’s identity, clustering it with NCBI sequences of *N. rosae,* and furthermore, positioning it in a distinct clade within *Neopestalotiopsis*, separated from *Pestalotiopsis*.

Although *Neopestalotiopsis* spp. are generally regarded as opportunistic pathogens that typically infect plants under stress conditions, *N. rosae* has demonstrated notably increased infection severity on strawberry, particularly in Florida, where symptoms are observed across multiple plant tissues [20]. In strawberry plants, *Neopestalotiopsis* leaf spot disease symptoms resemble those of leaf blotch (*Gnomoniopsis* spp.), scorch (*Diplocarpon* spp.), and typical leaf spots caused by other leaf spot pathogens [45,46]. However, leaf lesions caused by *N. rosae* are characterized by dark brown V-shaped necrotic areas starting at the leaf edge with well-defined borders and light brown centers [47]. Conidiomata are readily produced on the lesions, facilitating the spread of the pathogen.

In fruit tissues, the symptoms caused by *N. rosae* closely resemble those of anthracnose. However, a key distinguishing feature is the development of black spores on fruit lesions caused by *Neopestalotiopsis*, in contrast to the pinkish-orange-colored conidiomata produced by *Colletotrichum* spp. [48].

The symptoms observed in the infected strawberry plants, including necrotic leaf spots and water-soaked lesions on the fruits, align with those caused by *N. rosae* [16,20]. Although root rot caused by *N. rosae* has been described by other authors [15,23,27], root rot was not observed in the present study. Given the fact that the examined strain of *N. rosae* was virulent to various strawberry cultivars such as Aprica or Sengana under both field and greenhouse conditions, this strain is likely to be highly virulent. The symptoms directly impact the photosynthetic capability of the plants and reduce the marketability of the fruit, thereby threatening both yield and quality [49]. The high humidity and temperature conditions in the field likely facilitated the spread and severity of the disease, underscoring the importance of environmental factors in disease development [15].

This study highlights the early emergence and the accurate identification of *N. rosae* as a significant pathogen of strawberries, which is essential for the implementation of effective disease management strategies. Sequence data, particularly the combined analysis of ITS and TUB sequences, proved to be indispensable for the precise identification of *N. rosae*. This approach can be applied to other *Neopestalotiopsis* species to enhance our understanding of their distribution and impact on strawberry crops.

## 5. Conclusions

This study represents the first documented case of *N. rosae* causing leaf spot and fruit rot in strawberries across Europe including Germany, contributing valuable information to the understanding of the pathogen’s distribution and impact on strawberry production. Accurate identification of *N. rosae* through morphological, molecular, and pathogenic analyses is essential for understanding its role in strawberry disease dynamics. The observed virulence of *N. rosae* across various strawberry cultivars, combined with the impact of environmental conditions on disease severity, underlines the need for proactive and effective disease management strategies to limit its spread and economic impact. Ongoing monitoring and research into the distribution, pathogenicity, and ecological interactions of *N. rosae* will be essential to inform targeted control measures and preserve strawberry production across Europe and globally.

## Data Availability

Data are contained within the article; further inquiries can be directed to the corresponding authors.

## Appendix A

The taxa and sequences used for the phylogenetic analysis as well as the NCBI accession numbers for the isolated strains AETS11 and AETS11R are shown.

**Table A1:** Taxa and sequences of *Neopestalotiopsis* spp. and *Pestalotiopsis diversiseta* used in the phylogenetic analyses with NCBI accession numbers.

Taxa	Strain	Host	Country	ITS	β-Tubulin
*N. alpapicalis*	MFLUCC 17–2544a	*Rhizophora mucronata*	Thailand	MK357772	MK463545
	MFLUCC 17–2545	Symptomatic leaves of *Rhizophora apiculata*	Thailand	MK357773	MK463546
*N. aotearoa*	CBS 367.54a	Canvas	New Zealand	KM199369	KM199454
*N. acrostichi*	MFLUCC 17–1754a	*Acrostichum aureum*	Thailand	MK764272	MK764338
	MFLUCC 17–1755	*Acrostichum aureum*	Thailand	MK764273	MK764339
*N. australis*	CBS 114159a	*Telopea* sp.	Australia	KM199348	KM199432
*N. brachiata*	MFLUCC 17–1555a	*Rhizophora apiculata*	Thailand	MK764274	MK764340
*N. cubana*	CBS 600.96a	Leaf litter	Cuba	KM199347	KM199438
*N. egyptiaca*	PEST1	*Mangifera indica*	Egypt	KP943747	KP943746
*N. formicarum*	CBS 362.72a	Dead ant	Ghana	KM199358	KM199455
*N. honoluluana*	CBS 114495a	*Telopea* sp.	USA	KM199364	KM199457
*N. mesopotamica*	CBS 336.86a	*Pinus bruti*	Iraq	KM199362	KM199441
	CBS 299.74	*Eucalyptus* sp.	Turkey	KM199361	KM199435
*N. natalensis*	CBS 138.41a	*Acacia mollissima*	South Africa	NR_156288	KM199466
*N. piceana*	CBS 394.48a	*Picea* sp.	UK	KM199368	KM199453
	CBS 254.32	*Cocos nucifera*	Indonesia	KM199372	KM199452
	CBS 225.30	*Mangifera indica*	-	KM199371	KM199451
*N. protearum*	CBS 114178a	*Leucospermum cuneiforme* cv. “Sunbird”	Zimbabwe	JN712498	KM199463
	CMM1357	*-*	-	KY549597	KY549632
*N. rhizophorae*	MFLUCC 17–1550a	*Rhizophora mucronata*	Thailand	MK764277	MK764343
	MFLUCC 17–1551	*Rhizophora mucronata*	Thailand	MK764278	MK764344
*N. rosae*	CBS 101057a	*Rosa* sp.	New Zealand	KM199359	KM199429
	JZB340064	*Fragaria × ananassa*	China	MN495972	MN968336
	JZB340065	*Fragaria × ananassa*	China	MN495973	MN968337
	JZB340066	*Fragaria × ananassa*	China	MN495974	MN968338
	JZB340067	*Fragaria × ananassa*	China	MN495976	MN968339
	JZB340068	*Fragaria × ananassa*	China	MN495975	MN968340
	JZB340069	*Fragaria × ananassa*	China	MN495977	MN968341
	JZB340070	*Fragaria × ananassa*	China	MN495967	MN968342
	JZB340071	*Fragaria × ananassa*	China	MN495978	MN968343
	AETS11	*Fragaria × ananassa*	Germany	PQ511123	PQ584291
	AETS11R	*Fragaria × ananassa*	Germany	PQ511124	PQ621801
	CBS 124745	*Paeonia suffruticosa*	USA	KM199360	KM199430
*N. rosicola*	CFCC 51992a	*Rosa chinensis*	China	KY885239	KY885245
	CFCC 51993	*Rosa chinensis*	China	KY885240	KY885246
*N. saprophytica*	CBS 115452	*Magnolia* sp.	China	KM199345	KM199433
*N. sonneratae*	MFLUCC 17–1745a	*Sonneronata alba*	Thailand	MK764279	MK764345
	MFLUCC 17–1744	*Sonneronata alba*	Thailand	MK764280	MK764346
*N. surinamensis*	CBS 450.74a	Soil under *Elaeis guineensis*	Suriname	KM199351	KM199465
*N. thailandica*	MFLUCC 17–1730a	*Rhizophora mucronata*	Thailand	MK764281	MK764347
	MFLUCC 17–1731	*Rhizophora mucronata*	Thailand	MK764282	MK764348
*N. zimbabwana*	CBS 111495a	*Leucospermum cunciforme* cv. “Sunbird”	Zimbabwe	JX556231	KM199456
*Pestalotiopsis diversiseta*	MFLUCC 12–0287a	*Rhododendron* sp.	China	JX399009	JX399040

CBS: Centraalbureau voor Schimmelcultures, Westerdijk Fungal Biodiversity Institute, Utrecht, The Netherlands; JZB: Beijing Academy of Agricultural and Forestry Sciences, Beijing, China; MFLUCC: Mae Fah Luang University Culture Collection, Thailand.
